# Cross-ancestry proteome-wide Mendelian randomization prioritizes 12 plasma protein candidates for breast cancer risk

**DOI:** 10.64898/2026.05.04.26352055

**Published:** 2026-05-04

**Authors:** Xueyao Wu, Devika Godbole, Jacob Williams, Jayati Sharma, Jiyeon Choi, Zhonghua Liu, Peter Kraft, Haoyu Zhang

**Affiliations:** 1. Division of Cancer Epidemiology and Genetics, National Cancer Institute, Bethesda, MD, USA.; 2. Cancer Genomics Research Laboratory, Frederick National Laboratory for Cancer Research, Leidos Biomedical Research Inc, Rockville, MD, USA.; 3. Department of Biostatistics, Columbia University, New York, New York, USA.

**Keywords:** Plasma proteome, breast cancer risk, Mendelian randomization, polygenic score, proteogenomics, causal inference, genetic epidemiology

## Abstract

The plasma proteome provides a molecular bridge between genetic variation and disease risk, yet its contribution to breast cancer susceptibility across ancestries remains unclear. We conducted a proteome-wide Mendelian randomization (MR) study of 2,923 plasma proteins using *cis*-protein quantitative trait loci from 34,557 European participants in the UK Biobank Pharma Proteomics Project, integrated with genome-wide association studies of 156,901 breast cancer cases and 204,634 controls of European, East Asian, and African ancestries. Cross-ancestry meta-analysis identified 12 candidate proteins associated with breast cancer risk (*P* < 2.5×10^−5^), including six previously reported and six newly implicated in MR studies. DNPH1 showed cross-ancestry heterogeneity, with a risk-increasing association in European populations and a nominally inverse association in East Asian populations. CASP8, RALB, and USP28 displayed subtype-differentiated associations. Orthogonal validation provided variable support: six demonstrated strong evidence of statistical colocalization; four replicated in an independent European proteomic dataset (deCODE, n = 35,559); two replicated in an independent East Asian proteomic dataset (JCTF, n = 1,384); and four were supported by polygenic-score analyses in the ancestrally diverse All of Us cohort (9,250 cases, 214,857 controls). These findings prioritize a high-confidence subset of plasma proteins, including LRRC25, PARK7, and LRRC37A2, for future mechanistic and translational investigation.

## Introduction

Identifying the molecular mediators of breast cancer susceptibility across populations remains a central challenge in cancer biology. Although genome-wide association studies (GWAS) have identified over 230 genetic loci associated with breast cancer risk^1–8^, most risk-associated variants lie in noncoding regions, and their downstream effector mechanisms remain largely unknown^9^. The plasma proteome offers an informative intermediate layer for addressing this gap. Circulating proteins are closer to disease-related biology than germline variants, many are therapeutically tractable, and their levels can be anchored genetically through protein quantitative trait loci (pQTLs)^10–12^. Systematic mapping of genetically predicted protein levels onto breast cancer risk may therefore offer a route from GWAS signals to causal candidates, with direct implications for risk stratification and therapeutic target identification.

Mendelian randomization (MR) uses genetic variants as instrumental variables (IVs) to estimate the effect of an exposure, such as circulating protein levels, on disease risk^13,14^. Because germline variation is fixed at conception, MR is less susceptible than conventional observational analyses to confounding and reverse causation^15^, and genetically supported targets are more likely to succeed in drug development^14,16^. The potential of proteome-wide MR has expanded substantially with the UK Biobank Pharma Proteomics Project (UKB-PPP), which identified over 14,000 independent genetic associations for 2,923 plasma proteins using antibody-based profiling, representing roughly ~20-fold more protein associations than prior antibody-based studies^17^. These associations provide instruments of exceptional breadth and statistical strength to support systematic, proteome-scale causal inference for disease risk.

Previous MR studies of circulating proteins and breast cancer risk have provided valuable initial insights but faced notable limitations^18–28^. Most relied on older and smaller pQTL datasets with limited protein coverage, restricting both statistical power and biological breadth. Nearly all were conducted in European-ancestry (EUR) populations, limiting generalizability and the ability to assess whether genetically predicted protein effects are shared or heterogeneous across ancestries^29,30^. Breast cancer is also biologically heterogeneous: its intrinsic subtypes differ in molecular characteristics, prognosis, and likely etiology^7,31–33^, yet proteome-wide evidence across subtype contexts remains sparse.

**Figure 1.**
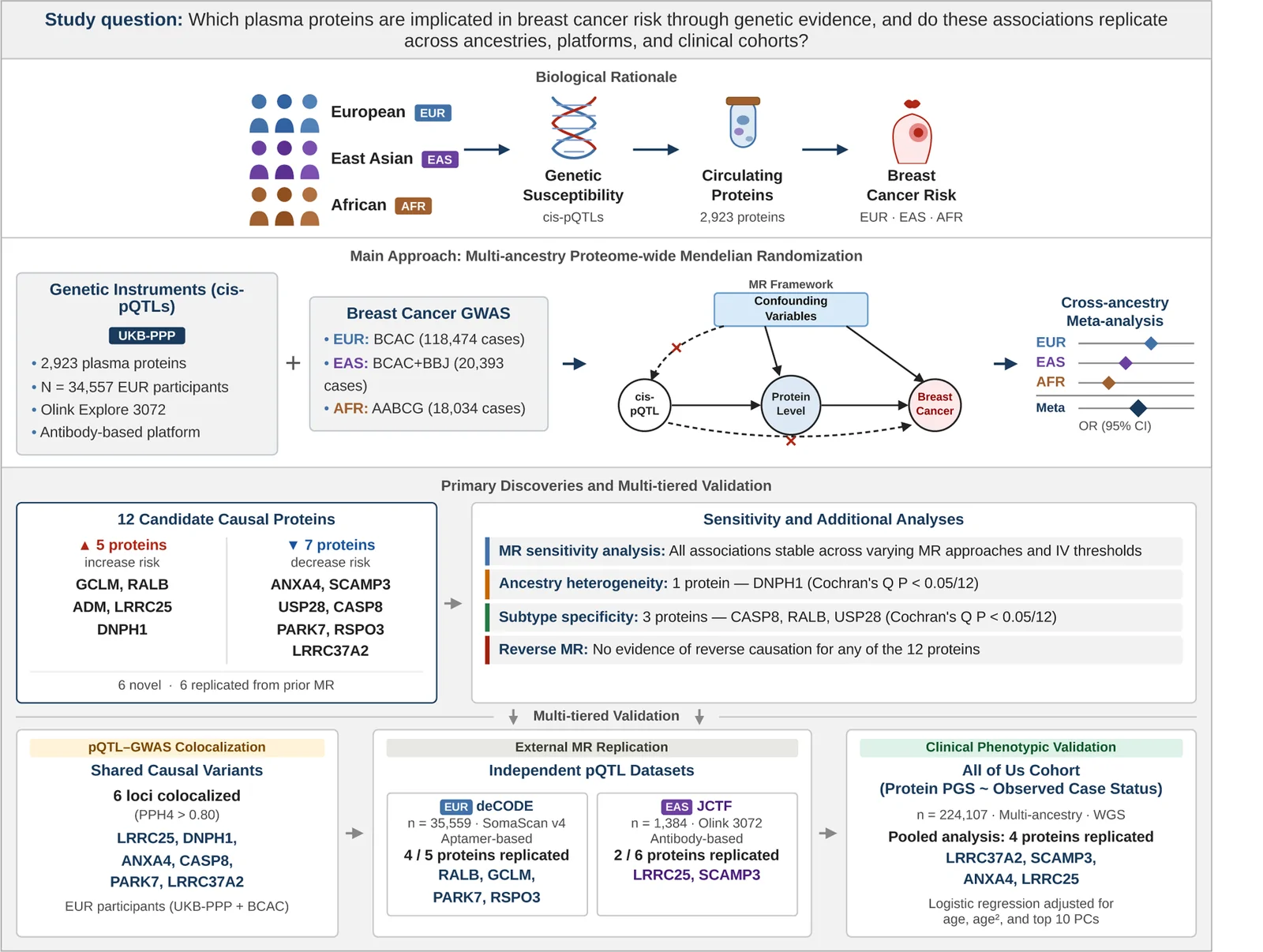
Graphical abstract. We conducted a proteome-wide Mendelian randomization (MR) study to evaluate associations between genetically predicted plasma protein levels and breast cancer risk. Genetic instruments derived from European-ancestry participants in the UK Biobank Pharma Proteomics Project (UKB-PPP; n = 34,557) were integrated with breast cancer genome-wide association studies (GWAS) from European, East Asian, and African ancestry populations (156,901 cases and 204,634 controls). Fixed-effects cross-ancestry meta-analysis identified 12 candidate proteins associated with breast cancer risk. These candidates were further evaluated using statistical colocalization, orthogonal MR replication in two independent proteomic datasets (deCODE, n = 35,559; Japan COVID-19 Task Force [JCTF], n = 1,384), and phenotype-linked genetic validation in the ancestrally diverse All of Us cohort (n = 224,107). Support varied across candidates, with a subset showing stronger multi-layer evidence for follow-up.

Here, we addressed these gaps through a proteome-wide MR analysis of breast cancer risk using *cis*-pQTLs for 2,923 plasma proteins from 34,557 EUR participants in UKB-PPP and breast cancer GWAS data from EUR, East Asian (EAS), and African (AFR) ancestry populations (156,901 cases and 204,634 controls in total)^6–8,17,34^. We combined ancestry-specific MR estimates through fixed-effects meta-analysis and prioritized 12 candidate proteins associated with overall breast cancer risk. We then evaluated subtype-specific associations and assessed robustness through a multi-tiered validation framework incorporating statistical colocalization, replication in independent European and East Asian pQTL resources^35,36^, and phenotype-linked genetic validation using protein polygenic scores in the ancestrally diverse All of Us cohort (AoU, n = 224,107)^37^. Together, these analyses prioritize plasma proteins linked to breast cancer risk and identify a subset with stronger genetic support for a putative causal role in breast cancer susceptibility. A graphical abstract is shown in Fig. 1.

## Results

### Proteome-wide cross-ancestry meta-analysis

Detailed characteristics of the pQTL and breast cancer GWAS datasets are provided in the Methods and Table 1. After harmonization with ancestry-specific breast cancer GWAS datasets and exclusion of proteins yielding no eligible *cis*-pQTLs, the final analyses included 2,061 proteins in EUR, 2,039 in EAS, and 2,031 in AFR ancestry datasets, corresponding to 2,044 unique proteins in the cross-ancestry meta-analysis. *Cis*-pQTLs for each protein, together with *F*-statistics and effect allele frequencies across the three ancestry-specific analyses, are listed in Supplementary Table 1. All selected *cis*-pQTLs had *F*-statistics > 10, indicating adequate instrument strength.

**Figure 2.**
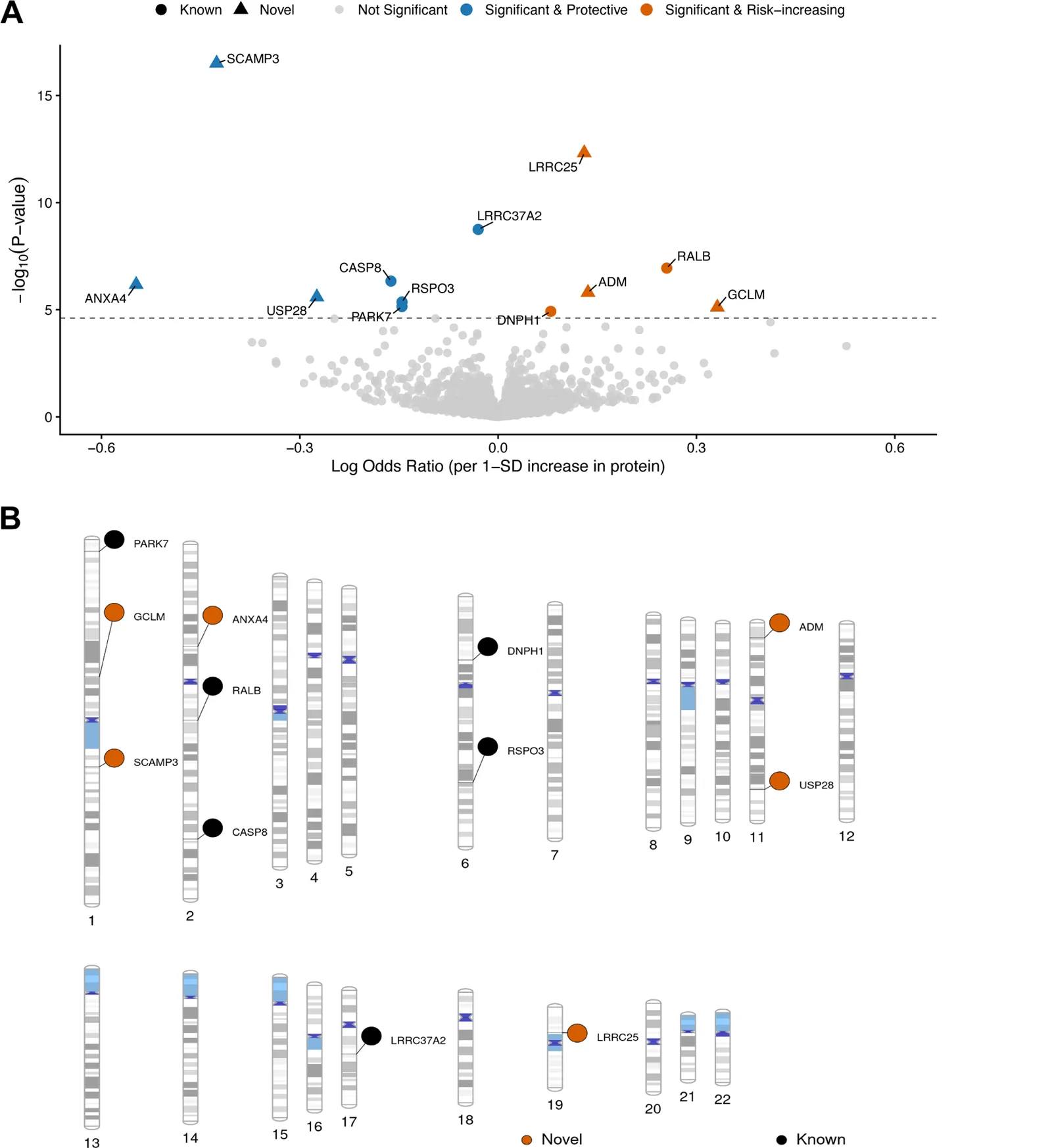
Volcano plot and genomic mapping of 12 candidate plasma proteins associated with overall breast cancer risk. (A) Volcano plot of cross-ancestry meta-analysis estimates for genetically predicted plasma protein levels in relation to overall breast cancer risk. The x-axis shows the log-transformed odds ratio (log OR) per 1-standard deviation increase in genetically predicted protein levels, and the y-axis shows statistical significance as the negative log_10_-transformed *P*-value. The horizontal dashed line indicates the Bonferroni-corrected significance threshold (*P* < 0.05/2,044). Of the 12 significant proteins, six replicated findings from prior MR studies (circles), and six were newly identified in the MR literature (triangles). Novelty is defined relative to prior MR literature and does not imply that these proteins have not been studied in breast cancer biology by other approaches. (**B**) Phenogram depicting the chromosomal locations of genes encoding the 12 candidate proteins. Previously reported proteins are shown in black, and newly identified proteins are shown in orange.

The primary inverse-variance weighted (IVW) cross-ancestry meta-analysis identified 12 plasma proteins associated with overall breast cancer risk after Bonferroni correction (*P* < 0.05/2,044) (Fig. 2a; Table 2). Of these, six proteins (RALB, DNPH1, CASP8, PARK7, RSPO3, LRRC37A2) corroborated previous MR findings^23,25–28^, whereas six (GCLM, ADM, LRRC25, ANXA4, SCAMP3, USP28) have not, to our knowledge, been previously implicated by MR in breast cancer risk.

Higher genetically predicted levels of five proteins were associated with increased breast cancer risk: GCLM (Odds Ratio [OR] per standard deviation [SD] increase = 1.39, 95% confidence interval [CI]: 1.20–1.61), RALB (OR = 1.29, 95% CI: 1.17–1.42), ADM ( OR = 1.15, 95% CI: 1.08–1.21), LRRC25 (OR = 1.14, 95% CI: 1.10–1.18), and DNPH1 (OR = 1.08, 95% CI: 1.04–1.12). Higher genetically predicted levels of seven proteins were associated with lower risk: ANXA4 (OR = 0.58, 95% CI: 0.47–0.72), SCAMP3 (OR = 0.65, 95% CI: 0.59–0.72), USP28 (OR = 0.76, 95% CI: 0.68–0.85), CASP8 (OR = 0.85, 95% CI: 0.80–0.91), PARK7 (OR = 0.86, 95% CI: 0.81–0.92), RSPO3 (OR = 0.86, 95% CI: 0.81–0.92), and LRRC37A2 (OR = 0.97, 95% CI: 0.96–0.98).

All 12 associations remained robust across seven complementary sensitivity analyses spanning alternative MR estimators (IVW, weighted-median, penalized IVW, and MR-Robust Adjusted Profile Score [MR-RAPS]) and relaxed instrument-selection thresholds (Supplementary Fig. 1; Methods), supporting robustness to horizontal pleiotropy and weak-instrument bias. Full primary and sensitivity-analysis results are provided in Supplementary Table 2. These 12 proteins were carried forward for downstream analyses, although the degree of orthogonal support varied across candidates.

### Ancestry-specific associations and cross-ancestry heterogeneity

**Figure 3.**
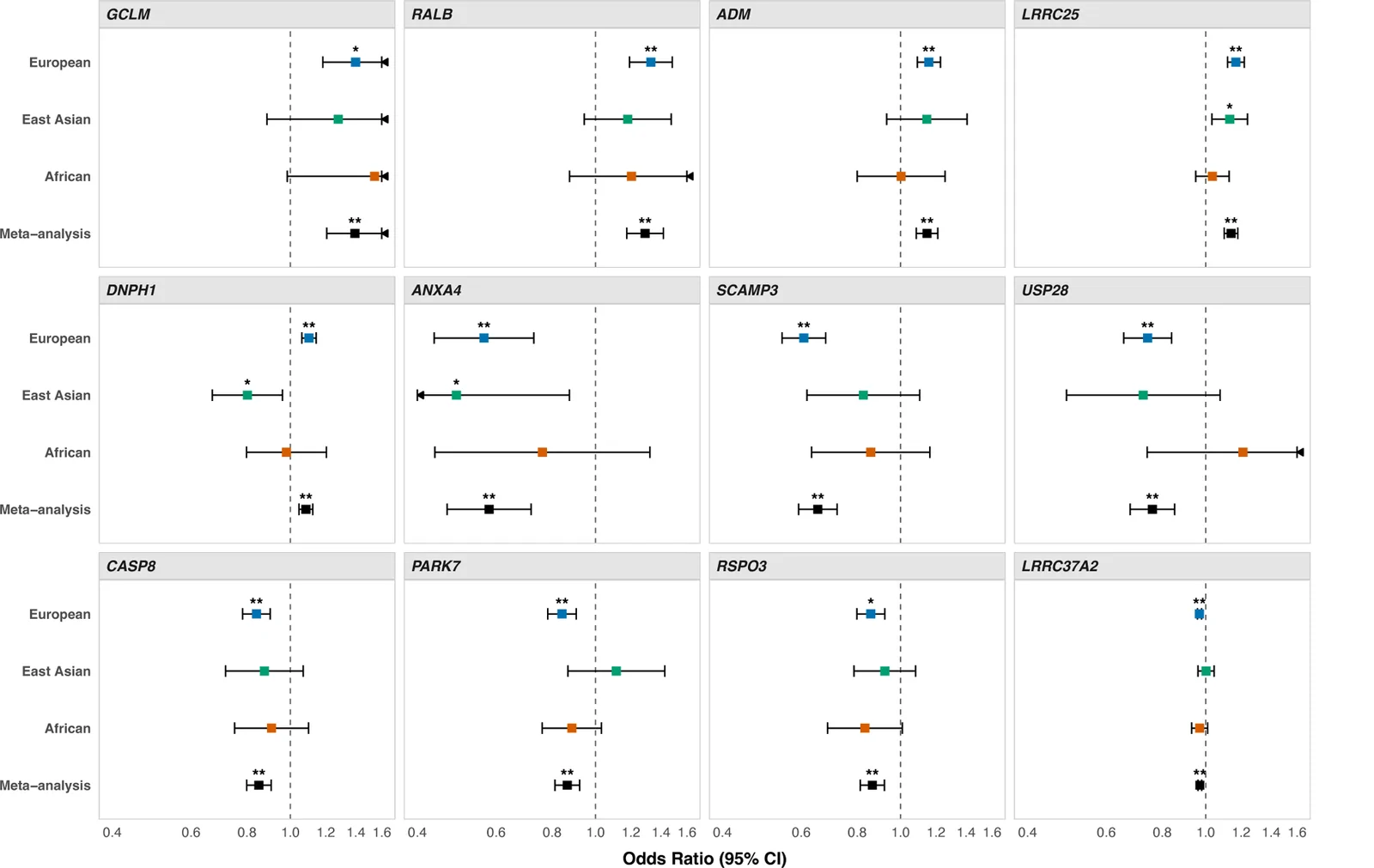
Forest plots of ancestry-specific and cross-ancestry MR estimates for 12 candidate plasma proteins in relation to overall breast cancer risk. Each point represents the odds ratio (OR) per 1-standard deviation increase in genetically predicted protein levels, and horizontal lines indicate 95% confidence intervals (CIs). Results are shown for the European, East Asian, and African ancestry-specific analyses and for the overall fixed-effects cross-ancestry meta-analysis. The x-axis is displayed on a log scale and truncated at OR = 0.4 and OR = 1.6 for visual clarity; arrows indicate CIs extending beyond this range. Double asterisks (**) denote Bonferroni-corrected significance, and single asterisks (*) denote nominal significance (*P* < 0.05).

Among the 12 proteins identified in the cross-ancestry meta-analysis, 10 (all except RSPO3 and GCLM) achieved Bonferroni-corrected significance in the EUR-specific analysis (*P* < 0.05/2,061), with effect directions consistent with the primary meta-analysis (Fig. 3; Supplementary Table 3). None of the 12 proteins reached Bonferroni-corrected significance in the EAS- or AFR- specific analyses, although LRRC25, DNPH1, and ANXA4 were nominally significant (*P* < 0.05) in EAS. This pattern probably reflects the substantially larger EUR breast cancer GWAS, together with potentially reduced transferability of EUR-derived pQTL instruments to non-EUR populations.

Evidence of cross-ancestry heterogeneity was observed only for DNPH1 (Cochran’s Q test *P* < 0.05/12). Higher genetically predicted levels of DNPH1 were associated with increased breast cancer risk in EUR (OR = 1.10, 95% CI: 1.05–1.14, *P* = 4.10×10^−07^), showed a nominally inverse association in EAS (OR = 0.80, 95% CI: 0.64–0.99, *P* = 1.63×10^−02^), and were null in AFR (OR = 0.98, 95% CI: 0.82–1.18, *P* = 0.85). To investigate whether this heterogeneity could be explained by EUR-derived exposure weights, we repeated the EAS analysis using *cis*-pQTL effect estimates from UKB-PPP EAS participants (n=262). Despite the limited sample size, the inverse association remained nominally significant and directionally consistent (IVW OR = 0.82, 95% CI = 0.70–0.97, *P* = 1.78×10^−02^). These results suggest that the directional difference is unlikely to be explained solely by EUR-derived effect estimates, although currently available non-EUR pQTL resources remain too limited to distinguish true biological heterogeneity from residual instrument limitations. Overall, ancestry-specific analyses indicate that most cross-ancestry signals were driven largely by EUR data, with DNPH1 representing the clearest exception.

### Associations with breast cancer intrinsic subtypes

**Figure 4.**
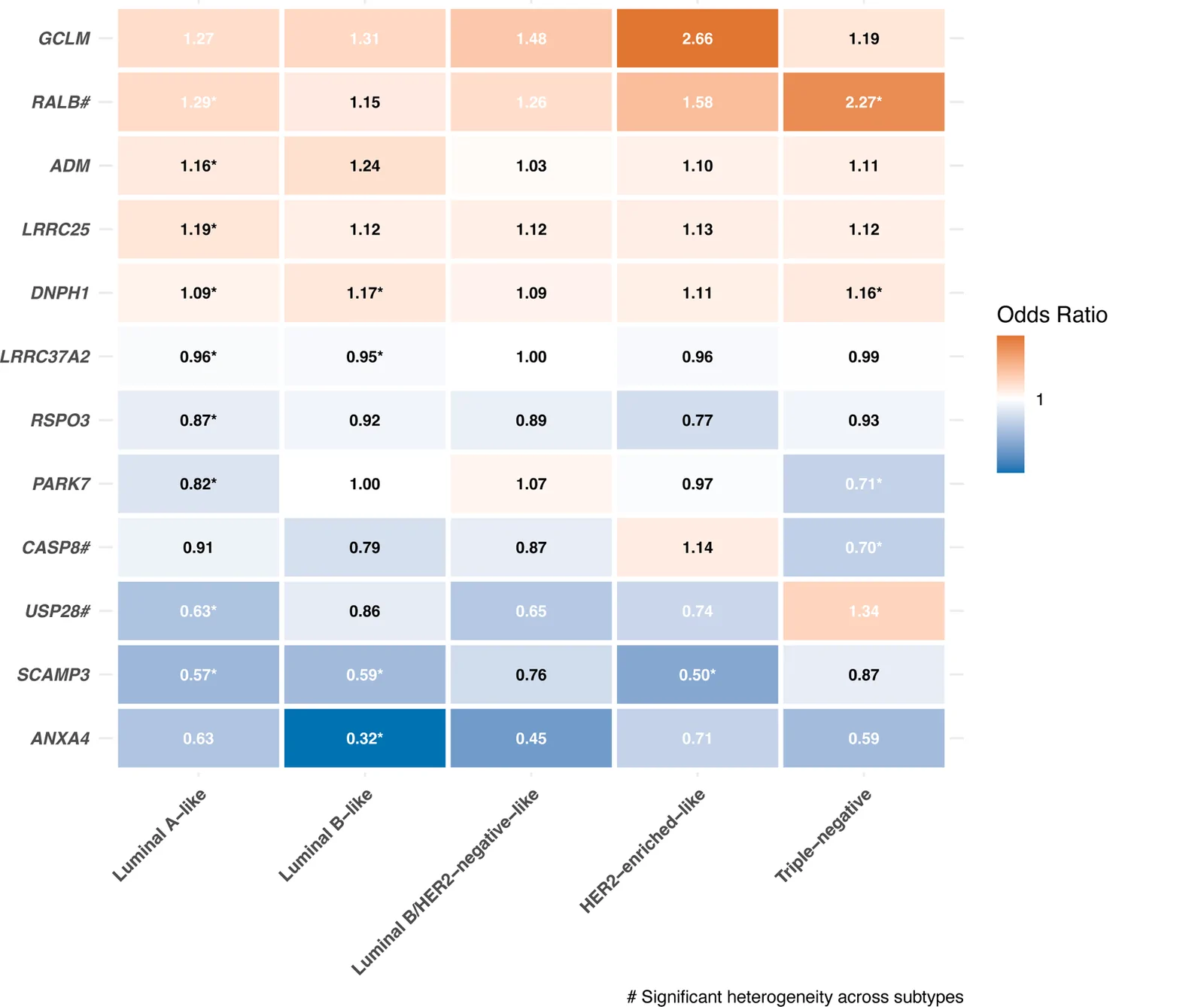
Heatmap of MR estimates for 12 candidate plasma proteins in relation to the risk of five intrinsic-like breast cancer subtypes in European-ancestry participants. Each tile represents the association between a given protein (y-axis) and breast cancer subtype (x-axis), derived from European-ancestry GWAS data. Tile color indicates the direction and magnitude of the odds ratio (OR): orange tiles indicate increased risk (OR > 1), and blue tiles indicate decreased risk (OR < 1); the value within each tile is the OR point estimate. Asterisks (*) indicate associations surviving Bonferroni correction for 12 tests (*P* < 0.05/12). A hash symbol (#) next to the protein name indicates significant heterogeneity of effects across subtypes by Cochran’s Q test, with Bonferroni correction for 12 proteins (*P* < 0.05/12). Apparent subtype-specific patterns for proteins without significant heterogeneity should be interpreted cautiously, given the smaller sample sizes available for several non-luminal A subtype analyses.

We next evaluated the 12 candidate proteins for association with five intrinsic-like breast cancer subtypes in EUR participants, defined jointly by estrogen receptor (ER), progesterone receptor (PR), and human epidermal growth factor receptor 2 (HER2) status (Methods; Fig. 4; Supplementary Table 4). After Bonferroni correction (*P*-value ≤ 0.05/12), 11 of the 12 proteins (all except GCLM) were associated with at least one subtype. Significant associations were most frequent for luminal A-like breast cancer (9 proteins), followed by luminal B-like (4), triple-negative (4), and HER2-enriched (1) subtypes. No protein reached Bonferroni-corrected significance for luminal B/HER2-negative-like disease. All significant subtype associations were directionally consistent with the corresponding overall breast cancer estimates.

Six proteins showed significant associations restricted to a single subtype: ADM, LRRC25, USP28, and RSPO3 with luminal A-like breast cancer; ANXA4 with luminal B-like breast cancer; and CASP8 with triple-negative breast cancer (TNBC). Five proteins demonstrated significant associations across multiple subtypes, including RALB and PARK7 (luminal A-like and TNBC), DNPH1 (luminal A-like, luminal B-like, and TNBC), SCAMP3 (luminal A-like, luminal B-like, and HER2-enriched), and LRRC37A2 (luminal A-like and luminal B-like). Because subtype-specific GWAS sample sizes differed substantially, these results should not be interpreted on their own as evidence of subtype specificity.

Three proteins showed statistically significant heterogeneity across subtypes after Bonferroni correction (Cochran’s Q test *P* ≤ 0.05/12): CASP8 and RALB had their strongest associations with TNBC, whereas USP28 showed its strongest association with luminal A-like disease (Fig. 4; Supplementary Table 4). These three proteins therefore provide the clearest evidence of subtype-differentiated effects, as their effect sizes differed significantly across subtypes and are less likely to reflect sample-size variation alone. For the remaining proteins, apparent between-subtype differences should be interpreted cautiously given the more limited sample sizes for several subtype-specific GWAS.

### Reverse-direction Mendelian Randomization

To assess whether the identified proteins were more consistent with upstream risk factors than with disease-reactive changes, we performed reverse MR using genetic liability to overall breast cancer as the exposure. We found no evidence that genetically predicted breast cancer risk influenced plasma levels of any of the 12 candidate proteins (all *P* > 0.10) (Supplementary Table 5).

### Multi-layer Validation

#### Colocalization analysis.

For each candidate protein, we performed colocalization analyses in EUR participants to evaluate whether a shared causal variant within the *cis*-regulatory region underlies both plasma protein levels and breast cancer risk. At a posterior probability of hypothesis 4 (PPH4) threshold of ≥ 0.80, six of the 12 candidate proteins showed strong evidence of colocalization: LRRC25, DNPH1, ANXA4, CASP8, PARK7, and LRRC37A2 (Table 2, Supplementary Table 6). Among these, four loci (LRRC25, DNPH1, CASP8, and LRRC37A2) showed SNP-level colocalization support (SNP PPH4 ≥ 0.80), nominating candidate causal variants for subsequent follow-up (Supplementary Table 6). The remaining six proteins (GCLM, RALB, ADM, SCAMP3, USP28, and RSPO3) did not meet the PPH4 threshold, raising the possibility that their MR associations reflect linkage disequilibrium (LD)-driven confounding of *cis*-pQTL instruments rather than evidence of a shared causal variant, warranting caution in their causal interpretation.

#### External replication in an independent European pQTL resource.

We next evaluated the 12 candidate proteins in the independent deCODE pQTL dataset (n = 35,559 Icelandic participants; SomaScan v4 aptamer-based platform)^35^, using deCODE-derived *cis*-pQTLs and the EUR breast cancer GWAS (Table 2). Of the five proteins available in deCODE, four replicated their EUR-specific MR associations at nominal significance (*P* < 0.05) with concordant directions: RALB and GCLM were associated with increased risk, while PARK7 and RSPO3 were associated with reduced risk.

Genetically predicted ANXA4 levels showed a significant positive association with breast cancer risk in deCODE (OR =1.15, 95% CI: 1.11–1.19, *P* = 3.71×10^−15^), directionally opposite to the inverse association obtained using UKB-PPP EUR pQTLs (OR =0.56, 95% CI: 0.44–0.73, *P* = 1.15×10^−05^). This discordance may reflect platform-dependent capture of different molecular features by aptamer- and antibody-based assays, and indicates that the direction of effect for ANXA4 remains uncertain.

#### External replication in an independent East Asian pQTL resource.

To assess EAS-specific support, we evaluated candidate proteins in the Japan COVID-19 Task Force (JCTF) pQTL dataset (n = 1,384 Japanese participants; Olink antibody-based platform)^36^, using JCTF-derived *cis*-pQTLs and the EAS breast cancer GWAS (Table 2). Given the small discovery sample, instrument availability and power were limited. Of the six candidate proteins available, two replicated their EAS-specific MR associations at nominal significance (*P* < 0.05) with concordant directions: LRRC25 (OR = 1.18, 95% CI: 1.05–1.32, *P* = 4.71×10^−03^) and SCAMP3 (OR = 0.75, 95% CI: 0.56–0.998, *P* = 4.83×10^−02^). For DNPH1, the single eligible instrument yielded a directionally consistent inverse association in EAS (OR = 0.93, 95% CI: 0.59–1.47, *P* = 0.75), though this did not reach nominal significance, most likely reflecting limited power rather than absence of association.

#### Phenotype-linked genetic validation in the All of Us cohort.

To evaluate whether inherited genetic predisposition to altered protein levels correlates with breast cancer risk in an independent multi-ancestry population, we constructed per-protein PGS and tested their associations against observed breast cancer status in the AoU cohort (9,250 cases and 214,857 controls pooled across five inferred genetic ancestries; Table 2, Supplementary Table 7). This analysis evaluates germline propensity linked to each protein rather than directly measured circulating protein levels.

In the pooled multi-ancestry analysis, four proteins showed nominally significant associations in the same direction as the primary MR estimates (*P* < 0.05): higher PGSs for LRRC37A2 (OR = 0.96, 95% CI: 0.94–0.98, *P* = 4.12×10^−05^), SCAMP3 (OR = 0.98, 95% CI: 0.96–0.998, *P* = 3.16×10^−02^), and ANXA4 (OR = 0.97, 95% CI: 0.95–0.99, *P* = 1.47×10^−02^) were associated with reduced breast cancer risk, whereas higher PGS for LRRC25 was associated with increased risk (OR = 1.03, 95% CI: 1.004–1.05, *P* = 1.83×10^−02^).

Ancestry-stratified PGS analyses yielded one additional directionally concordant finding: the protective association of USP28 was supported in the EAS stratum (OR = 0.83, 95% CI: 0.73–0.96, P = 9.26×10^−03^). ADM, which showed a null association in our primary AFR-specific MR, demonstrated a nominally significant inverse association in the AoU AFR stratum (OR = 0.93, 95% CI: 0.88–0.99, *P* = 1.34×10^−02^); this finding should be interpreted cautiously given the absence of AFR MR support.

#### Summary across validation layers.

The 12 candidate proteins differed in the extent of orthogonal support across validation analyses (Table 2). The strongest overall support was observed for LRRC25, PARK7 and LRRC37A2, each corroborated by the primary MR analysis, strong colocalization, and at least one external replication or phenotype-linked validation result. CASP8 and DNPH1 showed strong MR and colocalization corroboration, although external support was limited for CASP8 and interpretation of DNPH1 is complicated by cross-ancestry heterogeneity. RALB, GCLM, SCAMP3, RSPO3 and USP28 were supported by the primary MR analysis together with at least one additional validation layer, but lacked strong colocalization evidence. ADM and ANXA4 should be considered more provisional: ADM had limited support across validation analyses, whereas ANXA4 showed platform-dependent directional inconsistency.

## Discussion

This proteome-wide MR study identifies 12 plasma proteins associated with breast cancer risk and showed that support for these associations varies across ancestry-specific, subtype-specific, and validation analyses. By combining cross-ancestry MR with colocalization, external proteomic replication, and phenotype-linked genetic validation, we identified a subset of candidates with stronger cumulative evidence for involvement in breast cancer susceptibility. Collectively, these findings nominate a genetically prioritized subset of plasma proteins as promising candidates for mechanistic investigation and translational follow-up.

Our findings both reinforce prior work and extend it. Six proteins previously linked to breast cancer by MR were supported in this larger cross-ancestry analysis^23,25–28^, and six additional candidates emerged from the broader *cis*-pQTL coverage of UKB-PPP and the increased power and precision afforded by cross-ancestry meta-analysis. Colocalization further strengthened the causal evidence for six proteins, supporting shared genetic architecture within *cis*-regulatory regions that jointly influences both plasma protein levels and breast cancer susceptibility. The convergent lines of evidence increase confidence that these MR associations reflect genuine biological relationships rather than statistical artifacts, while those without strong evidence of colocalization warrant more cautious interpretation with respect to causal relevance^38^.

Among the more strongly supported candidates, CASP8, a key initiator of extrinsic apoptosis, has long been implicated in breast cancer susceptibility^39,40^. Transcriptome-wide association studies have linked higher genetically predicted CASP8 expression to reduced breast cancer risk in EUR and Asian populations, with nominal replication in AFR analyses^41–43^. LRRC25, a myeloid-enriched membrane protein, has recently been proposed as an immune checkpoint in solid tumors ^44^, and its elevated expression has been associated with immunosuppressive tumor microenvironments and worse prognosis^44,45^. LRRC37A2 has been implicated in recurrent chimeric RNAs identified in breast tumors that encode putative neoantigenic peptides, suggesting a possible link to tumor-specific transcriptional programs and immune recognition^46^. Other candidates, including ADM, SCAMP3, and USP28, also have biologically plausible roles through angiogenesis, membrane trafficking, and protein stability, respectively, and have been implicated in breast cancer cell proliferation or survival^47–51^. While these observations do not establish mechanism, they support the biological plausibility of the prioritized proteins.

A notable finding was the directionally heterogeneous association of DNPH1 across ancestries. This pattern could reflect true biological heterogeneity, but it could also arise from imperfect transferability of EUR-derived instruments to non-EUR populations, given ancestry differences in LD structure, allele frequency, and regulatory architecture. Two complementary analyses provided some evidence that EUR-derived effect estimates alone do not fully explain the directional difference: substituting EAS-specific *cis*-pQTL effect estimates preserved a nominally significant inverse association, and JCTF-derived instruments yielded a directionally consistent though non-significant result. Biologically, DNPH1 has been linked to the BRCA/PARP DNA repair pathway^52^, a critical axis with known population differences in founder mutations that could plausibly modulate its relevance across ancestries^53^. Whether such pathway-level variation underlies the observed heterogeneity or whether it reflects residual statistical uncertainty from underpowered non-EUR instruments cannot be resolved with currently available resources. More broadly, this result illustrates both the value and the current limitations of cross-ancestry proteogenomic inference, motivating the development of larger ancestrally diverse pQTL resources.

Subtype-stratified analyses further suggested etiological heterogeneity for a subset of proteins. Among the 12 candidates, CASP8, RALB and USP28 showed the clearest evidence of subtype-differentiated effects. Higher genetically elevated CASP8 levels were most strongly associated with reduced TNBC risk, consistent with experimental data linking CASP8 upregulation to enhanced apoptosis in TNBC cell lines^54,55^, and reinforcing the contribution of apoptotic dysregulation to TNBC susceptibility. RALB, an oncogenic small GTPase critical for TNBC cell viability^56,57^, similarly showed the strongest risk-increasing association in TNBC, further supporting a etiological role in this aggressive subtype. USP28 showed its most pronounced protective association with luminal A-like subtype alongside a positive, non-significant effect in TNBC. Given evidence that USP28 can stabilize oncogenic substrates and promote breast cancer cell survival^51,58^, these results suggest that its net effect may depend on tumor context. The five proteins with broader, subtype-spanning associations likely reflect effects on fundamental cellular processes common across breast cancer subtypes. Taken together, these findings underscore the molecular heterogeneity of breast cancer susceptibility and highlight the value of subtype-resolved proteomic MR evidence for guiding precision prevention and therapeutic prioritization.

Our external validations across independent datasets lend important support to a subset of findings. In deCODE, four of five testable proteins replicated using an independent aptamer-based proteomic platform, supporting the robustness of the associations for RALB, GCLM, PARK7, and RSPO3. ANXA4 showed a directionally opposite association in deCODE relative to UKB-PPP, consistent with previous reports that antibody- and aptamer-based assays can capture distinct molecular features and yield discordant genetically predicted disease associations^59,60^. Given the multiple isoforms and conformation-dependent membrane states of annexins^61^, platform-dependent capture of different molecular forms is a plausible explanation, although the direction of effect for ANXA4 remains uncertain. Replication of LRRC25 and SCAMP3 in the considerably smaller JCTF resource provides noteworthy, though still limited, evidence of cross-ancestry generalizability for these two proteins. PGS analyses in AoU replicated four proteins in the pooled analysis and one additional protein in ancestry-stratified analyses, supporting the generalizability of these associations in an ancestrally diverse cohort independent of the primary outcome GWAS. Because these analyses do not involve direct protein measurement, they complement rather than replace MR and colocalization evidence.

Our findings carry implications for mechanistic research and translational prioritization. The null reverse-direction MR results are more consistent with the prioritized proteins reflecting upstream risk-related processes than with purely disease-reactive biomarkers, although reverse MR alone cannot fully resolve biological directionality. Among the better-supported candidates, GCLM and DNPH1 encode enzymes with tractable pharmacological biology, and preclinical studies have described covalent targeting of glutamate cysteine ligase and DNPH1 inhibition in cancer models^52,62^. Our results for LRRC25 and LRRC37A2 provide genetically anchored rationale for exploring translational directions suggested by prior literature, including LRRC25 as a potential cancer immunotherapy checkpoint target and LRRC37A2-containing fusion transcripts as candidates for breast cancer vaccine development^44,46^. These translational hypotheses remain provisional and are most persuasive for the subset supported across multiple validation layers. Three priorities would advance this field: generation of large-scale ancestrally diverse pQTL resources to enable more robust non-EUR instrument discovery; prospective studies assessing protein-based risk prediction and early detection; and functional validation to establish the mechanistic roles of candidate proteins in breast cancer tumorigenesis.

Several limitations warrant consideration. First, the primary instruments were derived from EUR participants because comparably large non-EUR pQTL resources are not yet available. The cross-ancestry meta-analysis is therefore not a symmetric multi-ancestry analysis; it is more accurately characterized as a cross-ancestry outcome evaluation using predominantly EUR-derived instruments, with associations substantially driven by EUR data. We note that UKB-PPP reported strong concordance of *cis*-pQTL effect sizes across ancestry groups, providing empirical justification for our primary approach^17^. Second, our colocalization analyses assume a single causal variant per locus; in regions with allelic heterogeneity, this assumption may be violated, potentially reducing power or yielding conservative inference. Third, although the usage of *cis*-pQTLs minimize horizontal pleiotropy, some instruments may influence breast cancer risk through pathways partially independent of their target protein, and residual pleiotropy cannot be excluded entirely despite sensitivity analyses. Fourth, subtype-stratified analyses were restricted to EUR data and sex-stratified analyses were not possible given sex-combined pQTL data, though published evidence indicates strong consistency of cis-pQTL effects across sexes^63^. Fifth, MR estimates reflect lifelong genetically predicted protein levels and may not precisely reflect the consequences of pharmacological intervention at specific therapeutic time points.

In summary, this cross-ancestry proteome-wide MR study prioritized 12 plasma proteins associated with breast cancer risk and identified a higher-confidence subset supported by colocalization, external replication, and phenotype-linked genetic validation. These findings provide a genetically informed foundation for mechanistic investigation and highlight both the promise and the current limits of cross-ancestry proteogenomic inference in cancer epidemiology.

## Methods

### Overall Study Design

We conducted ancestry-specific two-sample MR analyses by pairing *cis*-pQTL instrument-protein associations derived from EUR participants in the UKB-PPP with instrument-breast cancer associations from large-scale GWAS in EUR, EAS, and AFR ancestry participants. Ancestry-specific MR estimates were subsequently combined via fixed-effects meta-analysis to generate cross-ancestry effect estimates. Fixed-effects meta-analysis was used as the primary discovery framework to identify proteins showing broadly consistent directional associations across ancestry-specific analyses, while Cochran’s Q statistic was used to test for cross-ancestry heterogeneity. Because sample size, LD structure, and instrument transferability differed across ancestries, pooled estimates were interpreted alongside ancestry-specific results. Proteins reaching proteome-wide significance in the cross-ancestry analysis were carried forward for downstream analyses, including subtype-specific MR, reverse-direction MR, statistical colocalization, external replication in independent pQTL datasets, and phenotype-linked genetic validation using PGS in the AoU cohort.

### Main Data Source

#### Protein Data Source

Genetic variant-protein association summary statistics were obtained from the UKB-PPP, which measured 2,923 plasma proteins using the antibody-based Olink Explore 3072 platform^17^ (Table 1). Our analysis utilized the discovery pQTL dataset from 34,557 EUR participants, broadly representative of the UKB cohort. Protein levels were measured as normalized protein expression (NPX) values on a log_2_ scale, then inverse-rank normalized and adjusted for key covariates, including age, sex, and genetic principal components, before genetic association testing. Genetic ancestry was defined by the UKB-PPP research group using pan-UKBB ancestry definitions. Although smaller AFR (n = 931) and EAS (n = 262) pQTL datasets were available within UKB-PPP, their sample sizes were insufficient for proteome-wide instrument discovery and were therefore used only in targeted sensitivity analyses for specific proteins.

#### Breast Cancer GWAS Data Sources

Breast cancer GWAS summary statistics were obtained from large, ancestry-stratified consortia (Table 1): the Breast Cancer Association Consortium (BCAC) for EUR ancestry (118,474 cases; 96,201 controls)^7^, a meta-analysis of BCAC and Biobank Japan (BBJ) for EAS ancestry (20,393 cases; 86,329 controls)^6,34,64^, and African Ancestry Breast Cancer Genetic Consortium (AABCG) for AFR ancestry (18,034 cases; 22,104 controls)^8^.

For proteins reaching proteome-wide significance, EUR-specific summary statistics for five intrinsic-like subtypes were additionally extracted from BCAC: luminal A-like (63,767 cases), luminal B/HER2-negative-like (15,942 cases), luminal B-like (15,942 cases), HER2-enriched-like (10,628 cases), and TNBC (8,602 cases), each compared against a common set of 91,477 controls^7^. Intrinsic-like subtypes were defined by the joint status of ER, PR, HER2, and tumor grade as follows: luminal A-like (ER+ and/or PR+, HER2−, grade 1 or 2); luminal B/HER2-negative-like (ER+ and/or PR+, HER2−, grade 3); luminal B-like (ER+ and/or PR+, HER2+); HER2-enriched-like (ER−, PR−, HER2+); and TNBC (ER−, PR−, HER2−).

### Mendelian Randomization Analysis

#### Data harmonization and instrumental variable selection.

EUR pQTL summary statistics were harmonized against each ancestry-specific breast cancer GWAS. Only variants present in both datasets were retained. Effect allele alignment was verified between datasets, and effect sizes were multiplied by −1 where necessary to align directions across overlapping variants. Within each harmonized variant set, *cis*-acting pQTLs were defined as variants within ±1 Mb of the protein-coding gene, excluding variants on sex chromosomes or those with imputation quality below threshold (INFO score < 0.3) or low minor allele frequency (MAF < 0.01). Independent *cis*-pQTLs were then identified via LD clumping (r^2^ ≤ 0.01 within a 2 Mb window) using the 1000 Genomes Project (1KGP) EUR reference panel^65^, retaining variants at a genome-wide significant threshold (*P* < 5×10^−8^) for primary analyses; relaxed thresholds used in sensitivity analyses are described below. Because harmonization was performed independently for each ancestry-specific GWAS, while LD clumping was applied consistently using the EUR reference panel, the final IV sets differ slightly across ancestry-specific analyses, reflecting variation in variant availability across GWAS datasets. Instrument strength was assessed via the *F*-statistic, and proteins yielding no eligible instruments after harmonization in a given ancestry were excluded from that analysis.

#### Primary and sensitivity MR analyses.

Primary analyses proceeded in two steps. First, ancestry-specific MR was conducted using the IVW method^13^ with instruments selected at *P* < 5×10^−8^. Second, ancestry-specific estimates were combined via fixed-effects meta-analysis to generate cross-ancestry effect estimates. Statistical significance was defined by a Bonferroni-corrected threshold based on the number of proteins tested (*P* < 0.05/2,044). Cross-ancestry heterogeneity was assessed using Cochran’s Q test, with the significance threshold Bonferroni-corrected for the number of significant proteins (*P* < 0.05/12).

Sensitivity analyses were conducted to evaluate robustness to horizontal pleiotropy and weak instrument bias, including the weighted median method^66^, which provides consistent estimates when up to 50% of instruments are pleiotropic; the penalized IVW method (pIVW)^67^, which corrects for weak instrument bias; and MR-RAPS^68^, which accounts for both systematic and idiosyncratic pleiotropy. Weighted median analyses used the same genome-wide significant instruments as the primary IVW analysis. pIVW and MR-RAPS analyses were additionally evaluated using relaxed IV selection thresholds (*P* < 5×10^−7^ and *P* < 5×10^−6^) to assess robustness to instrument selection criteria. An association was considered robust if it reached significance in the primary IVW meta-analysis and showed directionally consistent effects across all sensitivity methods when meta-analyzed across ancestries.

For DNPH1, which demonstrated significant cross-ancestry heterogeneity, a targeted sensitivity analysis was performed to assess whether the heterogeneity was attributable to EUR-derived instrument weights. EUR exposure estimates for the primary genome-wide significant instruments were replaced with EAS-derived *cis*-pQTL effect estimates (n = 262), and IVW MR was performed using these EAS-specific weights with the EAS breast cancer GWAS as the outcome.

#### Subtype-specific and reverse MR analyses.

For proteins with robust associations across primary and sensitivity analyses, additional MR analyses restricted to EUR participants were performed. For subtype-specific MR, the same pQTL instruments were applied, with subtype-specific breast cancer GWAS summary statistics substituted as the outcome; heterogeneity across subtypes was evaluated using Cochran’s Q test. For reverse MR, 277 independent genome-wide significant variants (*P* < 5×10^−8^) for overall breast cancer risk were identified from the EUR BCAC GWAS using the same LD clumping parameters as in the forward analyses and used as instruments, with corresponding SNP-protein associations extracted from the UKB-PPP EUR pQTL dataset. For all analyses, statistical significance was determined using a Bonferroni-corrected threshold based on the number of proteins tested.

All MR analyses were conducted in R using the MendelianRandomization, data.table, tidyverse, mr.raps, mr.pIVW, and meta packages.

### Multi-layer Validation

#### Colocalization analysis.

To strengthen causal inference, colocalization analyses were performed to test whether the observed MR associations were driven by a shared causal variant influencing both plasma protein levels and breast cancer risk. For each candidate protein, analysis regions were defined as ±150 kb windows around each lead *cis*-pQTL instrument. For proteins with multiple independent *cis*-pQTL instruments tested in separate genomic regions, colocalization was performed independently within each region, and the highest PPH4 value across regions is reported. Within each region, posterior probabilities for five mutually exclusive hypotheses (H0–H4) were calculated using the coloc R package with default prior probabilities^69^. Evidence for colocalization was evaluated based on PPH4, which quantifies support for a single shared causal variant; a PPH4 ≥ 0.80 in any tested region was considered strong evidence for colocalization^69^. To ensure accurate LD matching and reliable statistical inference, analyses were restricted to EUR ancestry data, using UKB-PPP EUR-specific summary statistics for protein levels and BCAC summary statistics for breast cancer.

#### External MR replication using independent pQTL resources.

External MR replication was performed for each candidate protein using independent pQTL datasets from deCODE (n = 35,559 Icelandic participants; SomaScan v4 aptamer-based platform)^35^ and JCTF (n = 1,384 Japanese participants; Olink Explore 3072 antibody-based platform)^36^ (Table 1). For each dataset, independent genetic instruments were identified using the same LD clumping procedure applied in the primary analysis, with ancestry-matched 1KGP reference panels used for LD estimation. Given the larger discovery sample in deCODE, a genome-wide significance threshold of *P* < 5×10^−8^ was applied for instrument selection; a relaxed threshold of *P* < 5×10^−7^ was used for JCTF to accommodate its more limited sample size. Instrument-outcome association estimates were extracted from the EUR and EAS overall breast cancer GWAS for deCODE and JCTF replication analyses, respectively, and IVW MR was performed using these instruments and their dataset-specific effect estimates. Supportive replication was defined as a directionally consistent association achieving nominal significance (*P* < 0.05).

#### Phenotype-linked genetic validation in the All of Us cohort.

To evaluate whether inherited genetic predisposition to altered levels of each candidate protein is associated with breast cancer risk in an independent, ancestrally diverse cohort, we constructed per-protein PGS and tested their associations with observed breast cancer status in AoU Controlled Tier v8^37^ (Table 1). Each PGS was computed as the allele-weighted sum of *cis*-pQTLs used in the corresponding EUR-specific primary MR analysis, with weights defined by their *cis*-pQTL effect estimates from UKB-PPP. Genetic data were derived from the whole-genome sequencing short-read SNP/indel ACAF-threshold release.

Breast cancer case status was defined using a curated set of OMOP condition_concept_id entries representing breast cancer-related diagnoses derived from breast-related malignant neoplasm concepts. Inferred genetic ancestry labels were used as provided by the AoU Research Program. Full details of the ancestry inference procedure are described in the AoU genomic data quality report. For cases, age at breast cancer diagnosis was assigned as the earliest age across qualifying condition records; controls retained their baseline age. Analyses were conducted in the multi-ancestry pooled sample and subsequently stratified by inferred genetic ancestry. Within each stratum, PGSs were standardized to unit variance before analysis. The association between each standardized PGS and breast cancer status was evaluated using logistic regression, adjusting for age, age squared, and the top 10 genetic principal components. Nominal significance (*P* < 0.05) was used as the replication threshold.

## Supplementary Material

Supplement 1

Supplement 2

## Figures and Tables

**Table 1.** Characteristics of datasets used in the analyses.

Study / Consortium	Ancestry	Sample Size (N or Cases/Controls)	Platform / Technology

Exposure Datasets (pQTLs)			
UKB-PPP (Discovery)	European	N = 34,557	Olink Explore 3072 (antibody-based)
deCODE (Validation)	European	N = 35,559	SomaScan v4 (aptamer-based)
JCTF (Validation)	East Asian	N = 1,384	Olink Explore 3072 (antibody-based)
Outcome Datasets (GWAS)			
BCAC	European	118,474 Cases / 96,201 Controls	OncoArray, iCOGS
BCAC & BBJ	East Asian	20,393 Cases / 86,329 Controls	OncoArray, Japonica Array v.2
AABCG	African	18,034 Cases / 22,104 Controls	Whole-genome sequencing, MEGA Array, OncoArray, iCOGS, and other arrays
Phenotypic Validation Dataset			
AoU (Validation)	Multi-ancestry	9,250 Cases / 214,857 Controls	Whole-genome sequencing (short-read SNP/indel ACAF-threshold release)

pQTL, protein quantitative trait locus; GWAS, genome-wide association study; UKB-PPP, UK Biobank Pharma Proteomics Project; JCTF, Japan COVID-19 Task Force; BCAC, Breast Cancer Association Consortium; BBJ, Biobank Japan; AABCG, African Ancestry Breast Cancer Genetic Consortium; AoU, All of Us Research Program.

**Table 2.** Summary of multi-tiered validation evidence for 12 candidate plasma proteins associated with breast cancer risk.

Protein	Cross-Ancestry OR (95% CI)^1^	Ancestry Heterogeneity (*P*-value)^2^	Coloc EUR PPH4^3^	deCODE EUR OR (95% CI)^4^	JCTF EAS OR (95% CI)^5^	AoU Pooled Multi-ancestry OR (95% CI)^6^

GCLM	1.39 (1.20–1.61)	0.82	0.22	1.11 (1.02–1.20)	N/A	1.01 (0.99–1.04)
RALB	1.29 (1.17–1.42)	0.58	0.00	1.15 (1.10–1.20)	N/A	1.001 (0.98–1.02)
ADM	1.14 (1.08–1.21)	0.50	0.47	N/A	N/A	1.002 (0.98–1.02)
LRRC25	1.14 (1.10–1.18)	4.75×10^−02^	1.00	N/A	1.18 (1.05–1.32)	1.03 (1.004–1.05)
DNPH1	1.08 (1.04–1.12)	2.15×10^−03^	0.97	N/A	0.93 (0.59–1.47)	1.02 (0.996–1.04)
ANXA4	0.58 (0.47–0.72)	0.52	0.89	1.15 (1.11–1.19)	1.002 (0.86–1.17)	0.97 (0.95–0.99)
SCAMP3	0.65 (0.59–0.72)	2.78×10^−02^	0.11	N/A	0.75 (0.56–0.998)	0.98 (0.96–0.998)
USP28	0.76 (0.68–0.85)	0.16	0.02	N/A	N/A	0.99 (0.97–1.01)
CASP8	0.85 (0.80–0.91)	0.72	0.90	N/A	N/A	0.99 (0.97–1.01)
PARK7	0.86 (0.81–0.92)	0.10	0.94	0.83 (0.75–0.91)	0.98 (0.72–1.33)	0.998 (0.98–1.02)
RSPO3	0.86 (0.81–0.92)	0.66	0.28	0.91 (0.87–0.94)	N/A	0.99 (0.97–1.01)
LRRC37A2	0.97 (0.96–0.98)	0.13	0.98	N/A	0.99 (0.95–1.02)	0.96 (0.94–0.98)

OR, odds ratio; CI, confidence interval; PPH4, posterior probability of hypothesis 4 (shared causal variant); N/A, protein not measured in the respective dataset or yielded no eligible *cis* -pQTLs after harmonization.

1 Cross-ancestry fixed-effects meta-analysis using pQTL instruments from the UK Biobank Pharma Proteomics Project (UKB-PPP) and breast cancer GWAS data from the Breast Cancer Association Consortium (BCAC), Biobank Japan (BBJ), and the African Ancestry Breast Cancer Genetic Consortium (AABCG).

2 *P*-value for cross-ancestry heterogeneity from Cochran’s Q test applied to the ancestry-specific MR estimates; Bonferroni-corrected significance was defined as *P* < 0.05/12.

3 Colocalization analysis using EUR summary statistics from UKB-PPP and BCAC. For proteins with multiple independent *cis*-pQTL regions tested, the highest PPH4 value is reported. PPH4 ≥ 0.80 was considered strong evidence for a shared causal variant.

4 MR using independent pQTL instruments from deCODE (n = 35,559 Icelandic participants; SomaScan aptamer-based platform) and EUR breast cancer GWAS data from BCAC. For RSPO3, results shown are for deCODE aptamer 8427_118. A second aptamer, 13094_75, yielded a consistent protective effect (OR = 0.90, 95% CI: 0.86–0.94).

5 MR using independent pQTL instruments from the Japan COVID-19 Task Force (JCTF; n = 1,384 Japanese participants; Olink antibody-based platform) and EAS breast cancer GWAS data from BCAC and BBJ. The small JCTF discovery sample limits instrument availability and statistical power; results should be interpreted accordingly.

6 Logistic regression of breast cancer case status on the standardized polygenic score for each protein in the multi-ancestry All of Us cohort (9,250 cases; 214,857 controls), adjusting for age, age squared, and the top 10 genetic principal components. This analysis evaluates genetic propensity linked to each protein and does not involve direct measurement of circulating protein levels.

## Data Availability

Summary-level GWAS statistics for plasma proteomics data are available through the UK Biobank (https://www.synapse.org/Synapse:syn51364943/), deCODE genetics (https://www.decode.com/), and the Japan COVID-19 Task Force (https://humandbs.dbcls.jp/files/hum0343/hum0343.v3.qtl.v1.zip). Summary-level GWAS statistics for breast cancer are available through https://www.ccge.medschl.cam.ac.uk/breast-cancer-association-consortium-bcac (European genetic ancestry), https://www.ccge.medschl.cam.ac.uk/breast-cancer-association-consortium-bcac/data-data-access/summary-results/gwas-summary-results, https://pheweb.jp/pheno/BrC (East Asian genetic ancestry), and https://www.ebi.ac.uk/gwas/studies/GCST90296719 (African genetic ancestry). Individual-level phenotype data and WGS data from the All of Us is available to registered researchers through the All of Us research workbench (https://workbench.researchallofus.org/).

## Code availability

Custom code used for data harmonization, Mendelian randomization analyses, colocalization, and validation analyses is publicly available at https://github.com/NCI-CGR/mr_breast_cancer.
