# Supplementary material for: Cross-ancestry proteome-wide Mendelian randomization prioritizes 12 plasma protein candidates for breast cancer risk
Source: medRxiv. 2026 May 4:2026.05.04.26352055. Preprint. [Version 1] doi: 10.64898/2026.05.04.26352055 (PMC13174725; doi:10.64898/2026.05.04.26352055)
Supplement: Supplementary file 1 [file media-1.docx]

**Supplementary Figure 1. Forest plots** **showing cross-ancestry associations between 12 candidate plasma proteins and overall breast cancer risk across sensitivity analyses.**

Each point represents the odds ratio (OR) per 1-SD increase in genetically predicted protein levels, with horizontal lines indicating 95% confidence intervals (CIs). The x-axis is displayed on a log scale and capped at OR values of 0.4 and 1.7 for visual clarity. Results are presented for seven Mendelian randomization sensitivity analyses using the following methods: inverse variance weighted (IVW), weighted median, penalized inverse variance weighted (pIVW), and MR-Robust Adjusted Profile Score (MR-RAPS). Genetic instruments were selected using three different *P*-value thresholds: *P* < 5×10^-8^, *P* < 5×10^-7^, and *P* < 5×10^-6^.
